# Enhanced Ductility of PEEK thin film with self-assembled fibre-like crystals

**DOI:** 10.1038/s41598-018-19537-1

**Published:** 2018-01-22

**Authors:** Yuan Wang, Binling Chen, Ken Evans, Oana Ghita

**Affiliations:** 1 School of Materials Science and Engineering, Sun Yat-sen University, Guangzhou, China; 2 University of Exeter, Department of Engineering, Mathematics and Physical Sciences, Exeter, Harrison Building, North Park Road, EX4 4QF United Kingdom

## Abstract

Poly Ether Ether Ketone (PEEK) is a high temperature polymer material known for its excellent chemical resistance, high strength and toughness. As a semi-crystalline polymer, PEEK can become very brittle during long crystallisation times and temperatures helped as well by its high content of rigid benzene rings within its chemical structure. This paper presents a simple quench crystallization method for preparation of PEEK thin films with the formation of a novel fibre-like crystal structure on the surface of the films. These quenched crystallised films show higher elongation at break when compared with conventional melt crystallised thin films incorporating spherulitic crystals, while the tensile strength of both types of films (quenched crystallised and conventional melt) remained the same. The fracture analysis carried out using microscopy revealed an interesting microstructure which evolves as a function of annealing time. Based on these results, a crystal growth mechanism describing the development of the fibre-like crystals on the surface of the quenched crystallised films is proposed.

## Introduction

**Figure 1:**
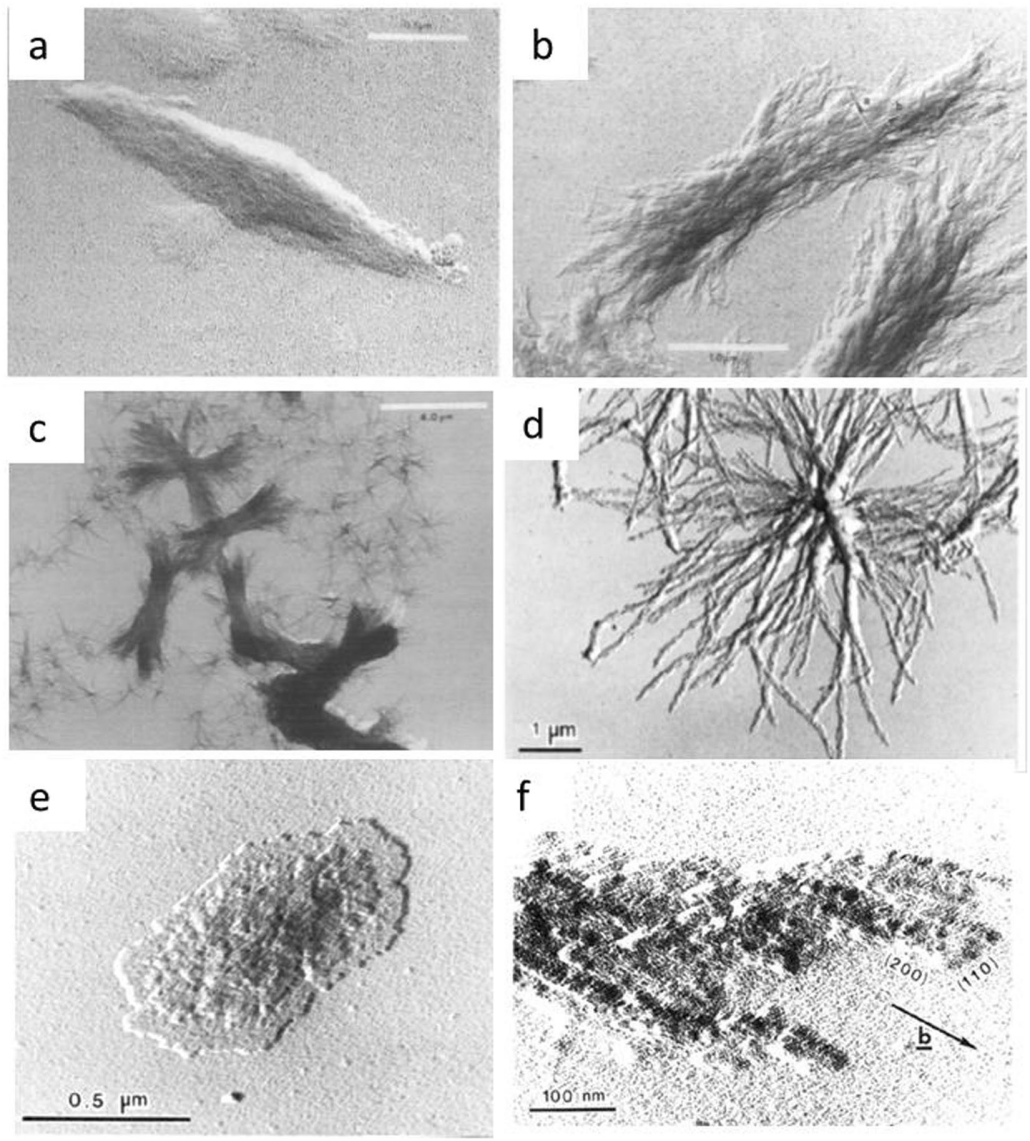
TEM micrograph of a Ryton V-I PPS (**a**) single crystal, (**b**) sheaf-like single crystal, (**c**) star-like single crystal aggregates of Ryton V-1 PPS^3^. (**d**) PEEK crystallized in α –chloronaphthalene solution, (**e**) PEEK single crystals grown from α-chloronaphthalene solution. The electron-diffraction patterns originate from the circled areas of the micrographs and are shown in correct orientation, (**f**) microfaceting in PEEK crystal^1^.

Semi-crystalline Poly Aryls such as PEEK, PolyEtherKetone (PEK) or PolyPhenyleneSulphide (PPS) demonstrate formation of various crystal morphologies depending on the crystallization condition^1–12^. PPS as well as PEEK received a lot of attention due to their excellent engineering performance at high temperatures. Shortly after their invention, several studies investigated the crystal morphology of solution grown PEEK and PPS thin films. Lovinger *et al*. prepared PEEK single crystals film from benzophenone and α-chloronaphthalene solution at elevated temperatures^1,2^. Microfaceting was observed in the growth of both single crystals and lamella, which has been explained as a disordered structure and fragmentation of crystals^1^. Similarly, Chung and Cebe reported various forms of single PPS crystals, needle-like PPS isolated single crystal, sheaf-like single crystal aggregates and star-like single crystal aggregates^3^ in thin films formed at elevated temperatures in dilute solutions of α- chloronaphthalene using a two-stage self-seeding technique. The self-seeding procedure includes a dissolution stage (PPS is dissolved in solution at specific temperature), first-stage isothermal crystallisation, seed generation stage, second-stage crystallisation, solution separation and replacement stage (uncrystallised PPS is removed from solution). The type of single crystal depends on seeding temperature and molecular weight. The typical morphology of PPS and PEEK solution growth crystals are shown in Fig. 1. Other investigations also identified the sheaf-like solution grown crystals by using single-stage crystallization method^4–7^.

**Figure 2:**
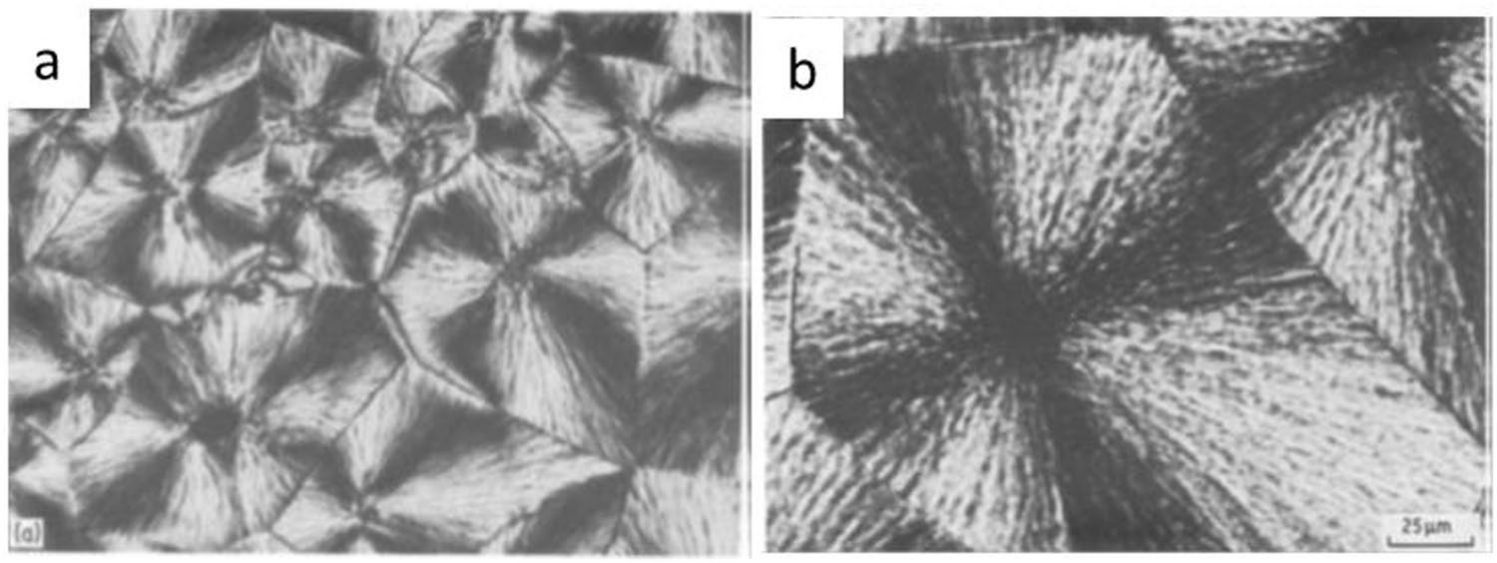
Photomicrographs of spherulites in crossed polars (**a**) PEEK and (**b**) PPS^10^.

The crystal morphology of PEEK and PPS from melt were also investigated^8–10^. The spherulites represent the most common crystal structure as demonstrated in Fig. 2.

**Figure 3:**
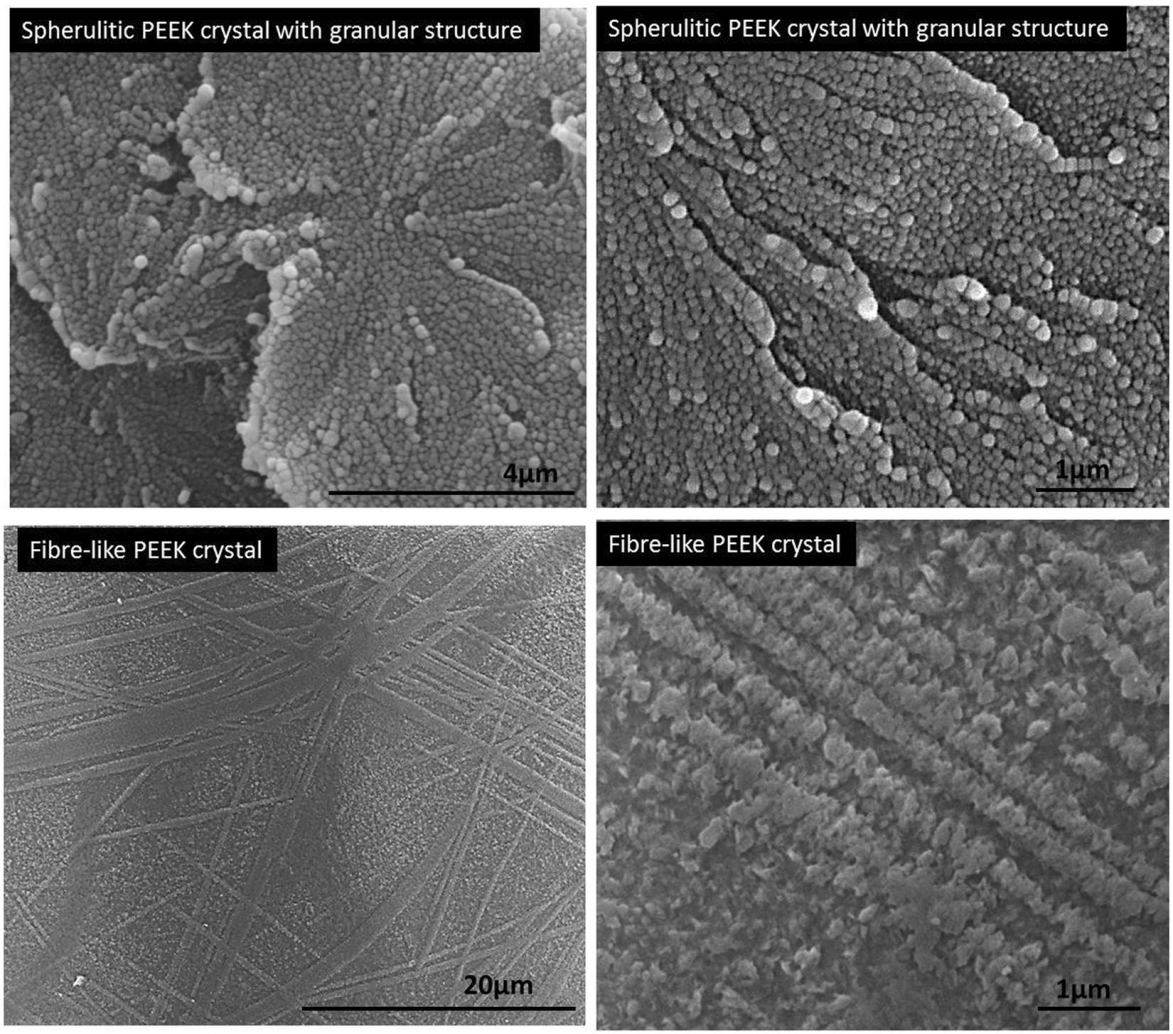
PEEK spherulitic crystals with granular structure developed in laser sintered bulk PEEK 150PF (top) and fibre-like crystals in PEEK 150PF thin films quenched crystallised and annealed at 300 °C for 120 mins (bottom)^11,12^.

Our previous study, on the morphology of spherulitic crystals in bulk PEEK samples manufactured by laser sintering and injection moulding techniques, found that spherulites consist of an hierachical granular crystal structure^12^. In a recent publication, we reported the formation of an unusual fibre-like crystal structure in PEEK thin films prepared by simple quench crystallization method for the first time^11^ (see Fig. 3) and suggested that these structures could have superior mechanical performance to the spherulitic PEEK films^12^.

Most of the studies on crystal structure of poly-aryls infer the effect such structures can have on polymer performance and only few papers made the link between structure, crystallinity and mechanical performance^13,14^. Although an excellent engineering material, PEEK has a low elongation at break in comparison with other polymers, especially when crystallised in a highly spherulitic structure. For example, HDPE has reported values of 400%^15^, nylon 12 has approximately 200%^16^ or nylon 6,6 has values of 90%^17^ in comparison with PEEK – e.g. PEEK 150PF has 15% elongation at break^18^. This study investigates the effect of the fibre-like crystals on the mechanical performance of PEEK thin films. Based on these results a crystal growth mechanism is proposed.

## Experiments

### Thin film preparation

Thin PEEK films were manufactured by melting and crystallization using two different methods. Method 1 allows fabrication of the conventional spherulitic crystals through melt crystallisation, used here as reference; and method 2 is used for fabrication of the novel fibre like crystals through quench crystallisation. The thickness of the film is approximately 250 µm. The thickness was controlled using an in-house built doctor blade rig.

**Method 1:** Firstly, the Victrex PEEK 150PF powder was spread evenly on a glass slide (Fisherbrand microscope slides, 0.8–1 mm thickness). No glass cover was applied on top of the PEEK powder. Then the powder layer was heated up on a hotplate (V14160 Bibby HC500 hotplate) at 400 °C for 5 mins. The molten film on the glass slides was quickly transferred to another hot plate and isothermal crystallized at 300 °C.

**Method 2:** Molten PEEK film was prepared in the same method as Method 1. To allow the formation of a transparent PEEK film, the molten film on the glass slide was immediately quenched in deionized water in this case. Then the transparent PEEK film was air dried at room temperature, followed by annealing on a hotplate at the temperature of 300 °C for 5, 10, 30, 60 and 120 mins.

### Scanning Electron Microscopy (SEM)

The surface of the thin PEEK films fabricated using the two crystallisation methods was examined by Scanning Electron Microscopy (SEM) (Hitachi S-3200N, Japan). The specimens were sputter coated with a 5 nm thick gold layer. The acceleration voltage was 20 kV.

### Tensile testing

The films prepared by the two film preparation methods were sliced into strips with the width of 3 mm and length of 6 cm. The gauge length of the strip is 2.5 mm. The tensile tests were performed on a Lloyds EZ20 machine with a crosshead speed of 20 mm min^−1^ for all samples. The test were repeated ten times for each type of sample.

### Thermal analysis DSC

Mettler Toledo DSC 821e/700 was used to carry out thermal analysis. The quenched crystallised and melt crystallised film samples were heated in DSC from 50 °C to 450 °C at a heating rate of 10 °C min^−1^ under nitrogen atmosphere at a flow rate of 50 ml min^−1^. The test were repeated three times for each type of sample. % crystallinity values of the PEEK films fabricated using the two crystallisation methods were calculated using the equation below:

1 $$\mathrm{Crystallinity}\,( \% )=\frac{{{\rm{\Delta }}{\rm{H}}}_{{\rm{melting}}}}{{{\rm{\Delta }}{\rm{H}}}_{100 \% }}\times 100$$

### X-Ray Diffraction (XRD)

The XRD investigation of films was measured by a Bruker D8 Advance XRD with copper anode at room temperature. XRD data were collected in the angular range where 2θ = 10°–40°. The step size of 2θ was 0.03°.

### Grazing Incidence X-ray Diffraction (GIXRD)

The GIXRD scan was collected with a grazing incidence angle of 1° by a Bruker D8 Advance XRD machine. 2θ was set as 10°–40°, and the step size of 2θ was 0.03°.

### Transmission Electron Microscopy (TEM)

The film samples were embedded into epoxy resin and allowed to cure before slicing through the thickness of the PEEK film. TEM samples with thicknesses of approximately 100 nm were sectioned in a microtome (Ultracut, Reichert-Jung, USA) from the pyramidal tip. The TEM specimens in cross-section area were placed on copper grids for analysis. The TEM images were captured using a JEM 2100 (JEOL, Japan) at acceleration voltage of 100 kV.

## Result and Discussion

**Figure 4:**
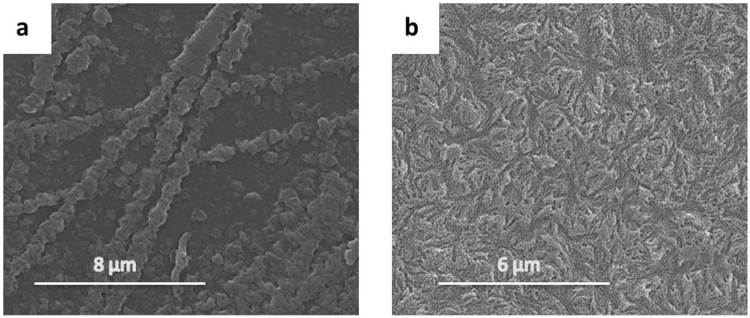
The presence of (**a**) fibre like crystal in the quench crystallized PEEK film and (**b**) conventional spherulitic crystal in melt crystallized PEEK film.

In our previous publication^11^ we discussed the formation of fibre like crystal structures in PEEK film by simple quench crystallization. Figure 4(a) and (b) shows the morphology of fibre like crystal and conventional spherulitic crystal, respectively.

**Figure 5:**
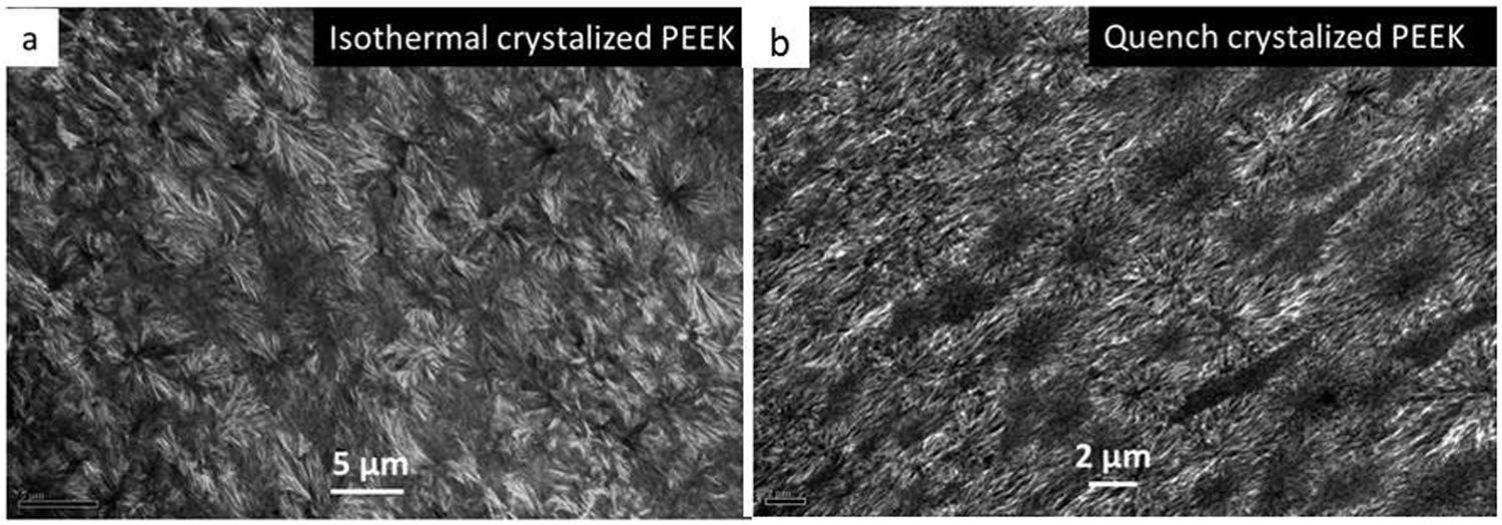
TEM images of (**a**) melt crystallized PEEK and, (**b**) quenched crystallised PEEK cross-sections.

Figure 5 shows TEM cross-section images of melt crystallised and quenched crystallised films with a typical PAEKs spherulitic morphology as reported in other studies^12^. The TEM cross-sections of the films present similar crystal morphologies independent of the crystallisation process, which suggest that the fibrils are a surface feature rather than an in-depth morphology. The AFM results of these fibre-like crystals, presented by the same authors in a previous study^11^, seems to lead to a similar conclusion. Based on these observations, XRD and Grazing Incidence XRD (GIXRD) had been employed in order to determine if there are any changes in crystal structure between the depth of the film and the surface of the film.

**Figure 6:**
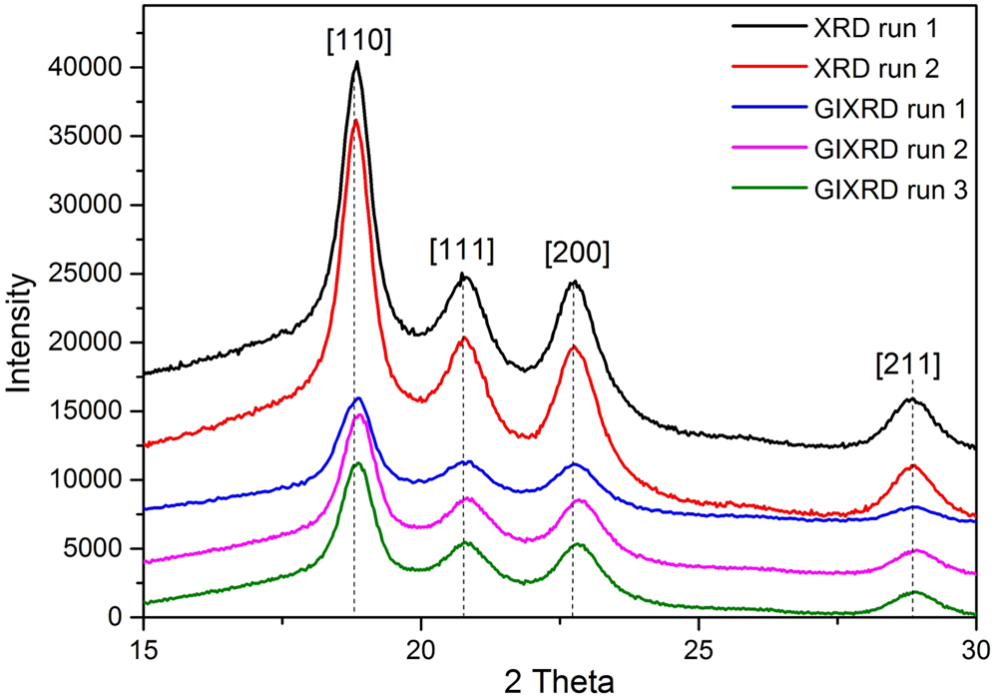
XRD and GIXRD spectra of quenched crystallised PEEK films annealed for 120 mins.

The XRD and GIXRD spectra of quenched crystallised PEEK films annealed for 120 mins are presented in Fig. 6, Tables 1 and 2. In Figure 6, the peaks at 18.8°, 20.8°, 22.8°, and 28.9° are attributed to the PEEK crystal planes [110], [111], [200], [211], respectively^11^. Based on the peak position of crystal planes and the full width at half maximum intensity (FWHM), the crystal thicknesses were calculated. The crystal thickness of the [110] peak was slightly smaller in the surface structure than in the depth of the sample. This was the most difference noticed between the surface and the depth of the crystal structure.

**Table 1:** Crystal thicknesses measured and calculated from the surface of the quenched crystallised films annealed for 120 mins using the grazing incidence XRD at a diffraction angle of 10–40°.

Peaks	Grazing Incidence XRD (GIXRD) of quenched crystallised PEEK film annealed for 120 mins								
	Run 1			Run 2			Run 3		
	2θ	FHWM	Crystal Thickness (nm)	2θ	FHWM	Crystal Thickness (nm)	2θ	FHWM	Crystal Thickness (nm)
[110]	18.9	0.704	12.7	18.9	0.802	11.2	18.8	0.707	12.6
[111]	20.8	0.934	9.6	20.8	1.214	7.4	20.8	0.958	9.4
[200]	22.8	0.933	9.6	22.8	1.031	8.7	22.8	0.940	9.5
[211]	28.8	1.164	7.8	28.7	0.998	9.1	28.7	0.998	9.1

**Table 2:** Crystal thicknesses measured and calculated from the depth of the quenched crystallised films annealed for 120 mins using the conventional XRD method at a diffraction angle, 2θ, varying between 10 and 40°.

Peaks	XRD – Quenched crystallised PEEK film annealed for 120 mins					
	Run 1			Run 2		
	2θ	FHWM	Crystal Thickness (nm)	2θ	FHWM	Crystal Thickness (nm)
[110]	18.7	0.648	13.8	18.7	0.669	13.4
[111]	20.6	0.889	10.1	20.6	1.391	6.4
[200]	22.5	0.913	9.8	22.5	1.062	8.5
[211]	28.5	1.123	8.1	28.6	1.168	7.8

**Figure 7:**
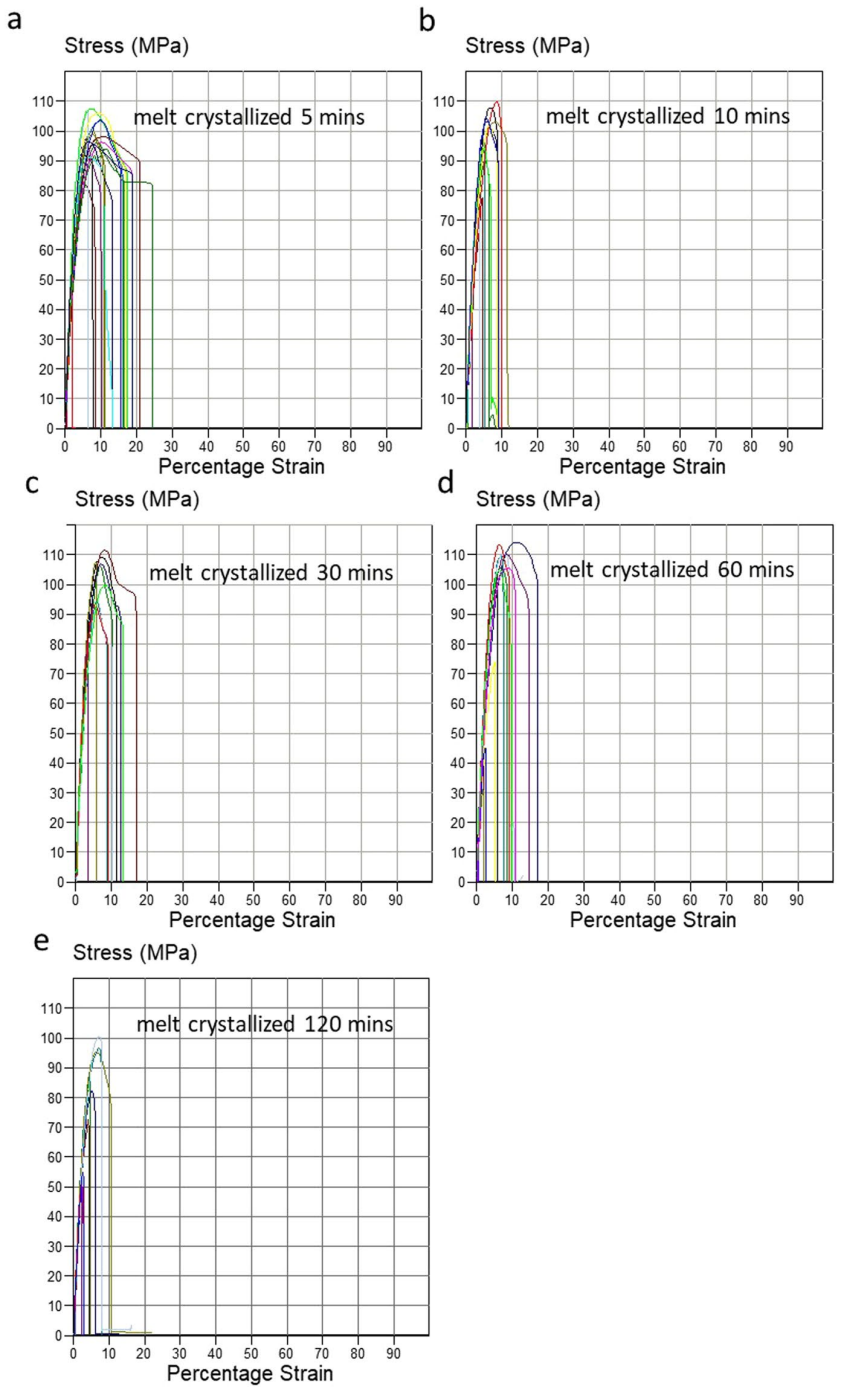
The tensile testing curves of melt crystallized PEEK film with various crystallization times (**a**) 5 mins, (**b**) 10 mins, (**c**) 30 mins, (**d**) 60 mins and (**e**) 120 mins (10 repeat measurements for each crystallisation time).

**Figure 8:**
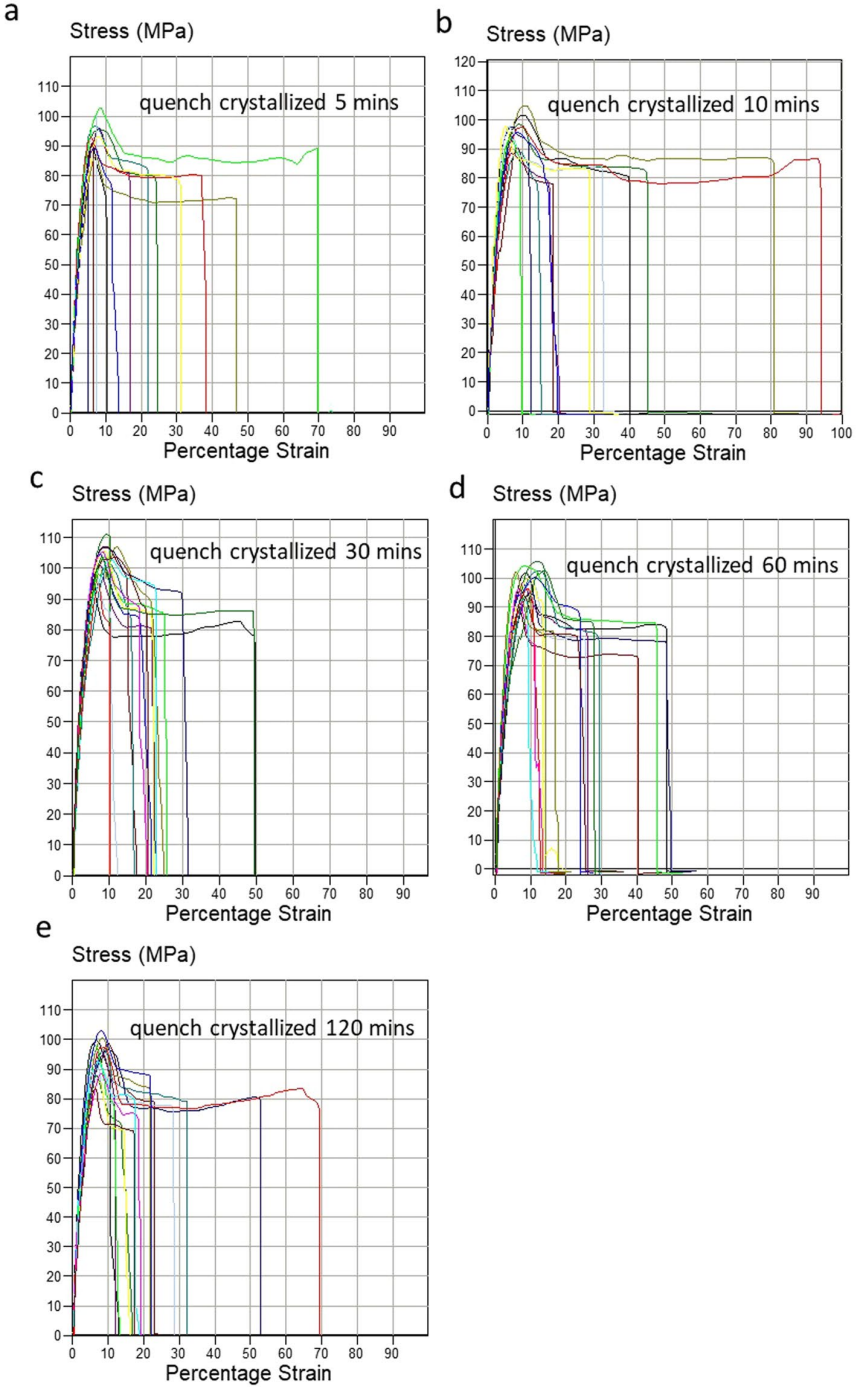
The tensile testing curves of quench crystallized PEEK films with various crystallization times (**a**) 5 mins, (**b**) 10 mins, (**c**) 30 mins, (**d**) 60 mins and (**e**) 120 mins (10 repeat measurements for each crystallisation time).

The stress-strain profiles of the films made with the two crystallization methods at different crystallisation times (5, 10, 30, 60 and 120 mins) are illustrated in Figs 7 and 8 and their mechanical data is summarised in Table 3. The film with fibre like crystals shows increased ductile fracture behaviour compared with the melt crystallized samples, though the variation of elongation at break amongst quench crystallized samples is relatively large. As it can be seen in Fig. 4(a), the fibre-like crystals are randomly distributed across the surface, therefore it is possible that the elongation at break values are dependent on the fibres orientation across each sample tested. It can be assumed that when fibres are predominantly orientated along the length of the sample (the testing direction), the elongation at break is increased. However, further investigation is required to confirm this assumption. Table 3 shows that the maximum elongation at break values achieved are as high as 70–90% for fibre like crystal films, 3.5–4.5 times higher than the ones for the melt crystallized films of only 20% elongation.

**Table 3:** Tensile properties of PEEK film with spherulites and fibre like crystals, (a) yield strength and (b) elongation at break.

Annealing time	Ultimate Tensile Strength (MPa)	
	Melt crystallisation	Quench Crystallisation
5 min	85 ± 5	80 ± 5
10 mins	94 ± 7	85 ± 4
30 mins	88 ± 5	89 ± 4
60 mins	96 ± 9	82 ± 5
120 mins	77 ± 11	80 ± 6
**Annealing time**	**Yield Strength (MPa)**	
	**Melt crystallisation**	**Quench Crystallisation**
5 min	95 ± 10	95 ± 10
10 mins	98 ± 10	98 ± 10
30 mins	102 ± 10	103 ± 10
60 mins	105 ± 5	100 ± 10
120 mins	78 ± 14	98 ± 5
**Annealing time**	**Elongation at break %**	
	**Melt crystallisation**	**Quench crystallisation**
5 min	5–20	10–50
10 mins	5–10	10–90
30 mins	5–15	10–50
60 mins	5–15	10–50
120 mins	5–10	10–70

The increase in elongation at break in the quenched crystalised films is the result of extended strain following the yield point. This behaviour was noticed in approximately 50% of the quenched crystallised samples in Fig. 8, in comparison with the melt crystallised films which break immediately after the yield point. The extended strain beyond the yield point is representative of neck formation.

**Figure 9:**
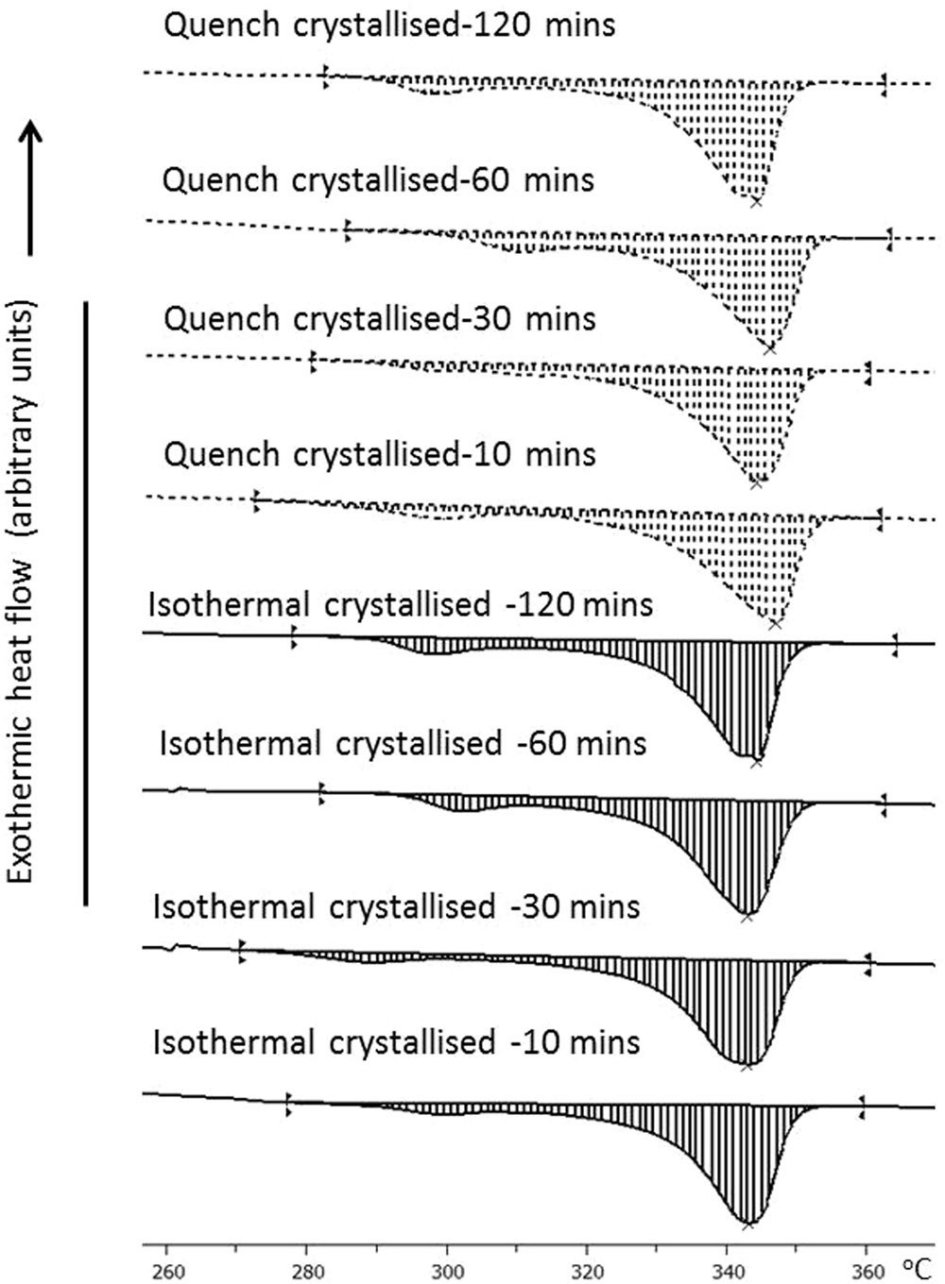
The DSC traces of PEEK film crystals under various crystallization conditions.

The mechanical performance of the films can be influenced by the morphology of the crystals and the degree of crystallisation. The SEM study has shown that the fibre-like crystals vary in orientation and thickness. However it is not clear whether the degree of crystallinity has been affected as well and therefore contributes to the changes noticed in the tensile strength data. For this reason, the crystallinity of quench and isothermal crystallised samples was examined by DSC. The DSC traces of quench crystallised and isothermal crystallized samples in Fig. 9 show the typical double melting peaks of PEEK^19^. The crystallinities of the quenched and isothermally crystallized samples are presented in Table 4. No significant difference in crystallinity was noticed between the two crystallisation methods used and the associated annealing times. This suggests that the crystal morphology is the significant factor influencing the elongation at break in the quenched crystallised samples instead of crystallinity.

**Table 4:** The crystallinity of the PEEK film samples fabricated by the two different crystallisation methods at 10, 30, 60 and 120 mins annealing times.

	Annealing time	Average Crystallinity (%) ± STD
Melt Crystallised	10 mins	38.1 ± 0.5
	30 mins	38.6 ± 0.9
	60 mins	38.3 ± 0.9
	120 mins	39.2 ± 0.3
Quenched Crystallised	10 mins	40.5 ± 4.2
	30 mins	38.5 ± 1.2
	60 mins	37.3 ± 1.3
	120 mins	38.6 ± 0.6

**Figure 10:**
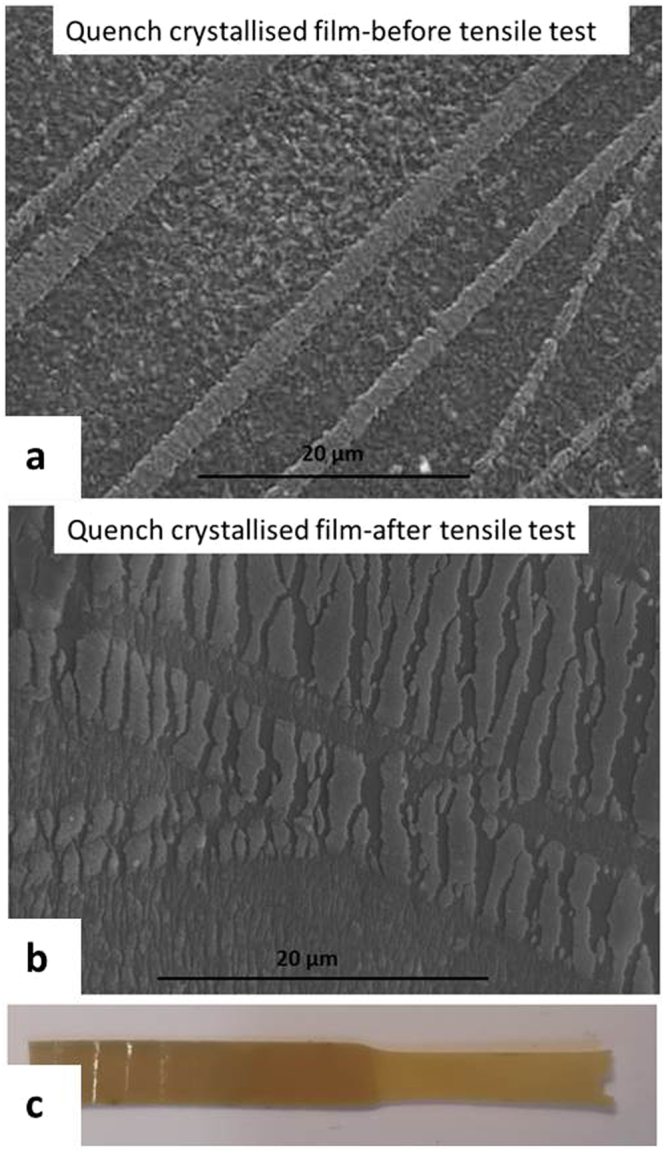
SEM images of the quench crystalised PEEK films (annealed for 60 mins) showing the changes suffered by the fibre-like crystals during the tensile test (**a**) before test (**b**) after test (**c**) the necking region after tensile test.

**Figure 11:**
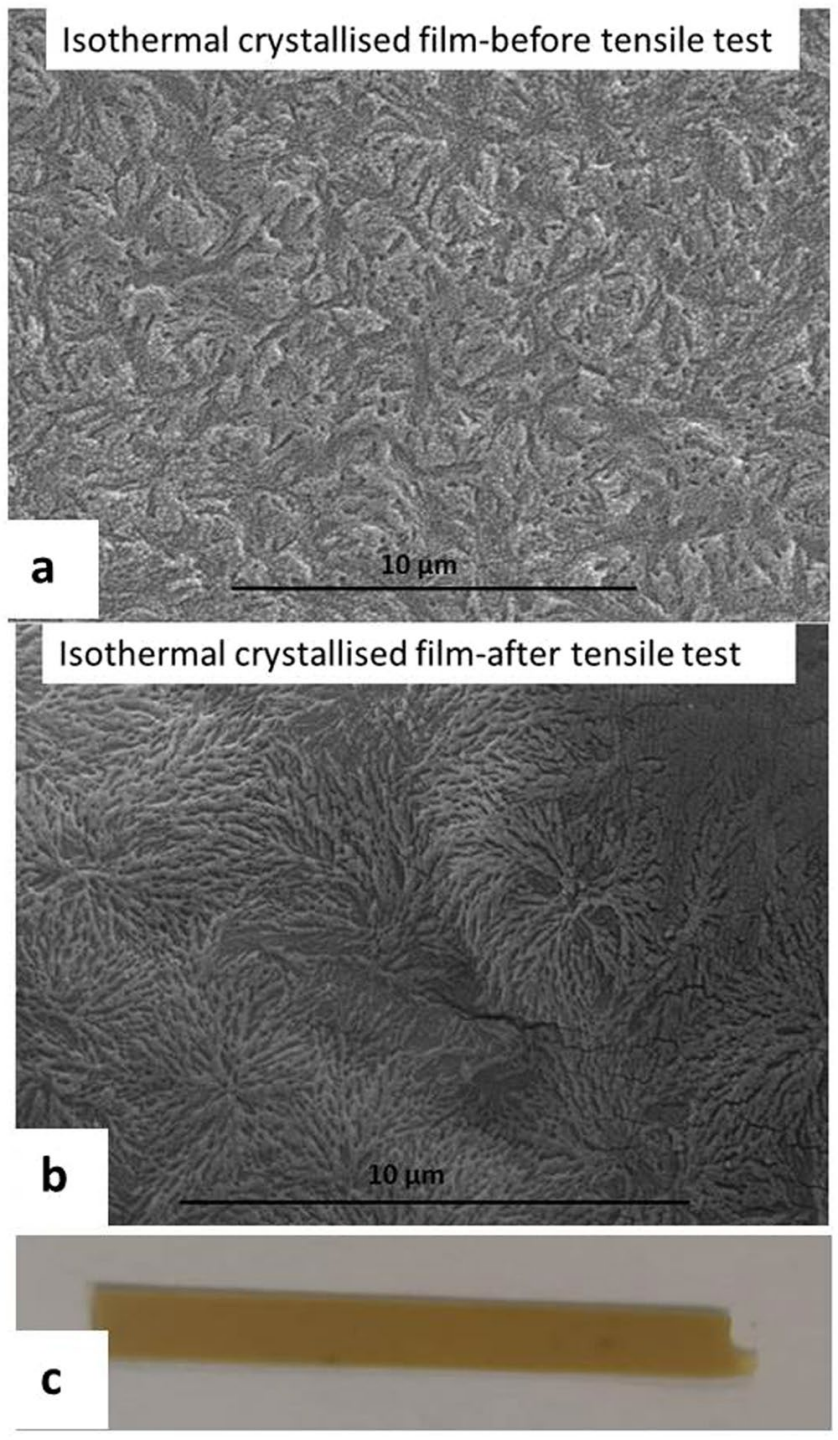
SEM images of the melt crystalised PEEK films (annealed for 5 mins) showing the changes suffered by the spherulites during the tensile test (**a**) before test (**b**) after test (**c**) the necking region after tensile test.

In the previous study, it was found that the width of the fibre like crystals increases with crystallization time^11^. Although it is difficult to further relate the crystal width with the tensile property of the quench crystallized film, a close examination of the film surfaces (post testing) revealed further details related with the growth of these fibres. Figure 10 presents the quenched crystallised film annealed for 60 mins before and after tensile testing as well as an optical image of the tested film with a visible neck region. Figure 11 shows the melt crystallised film annealed for 5 mins before and after tensile testing and an optical image of the tested film with a brittle failure end.

**Figure 12:**
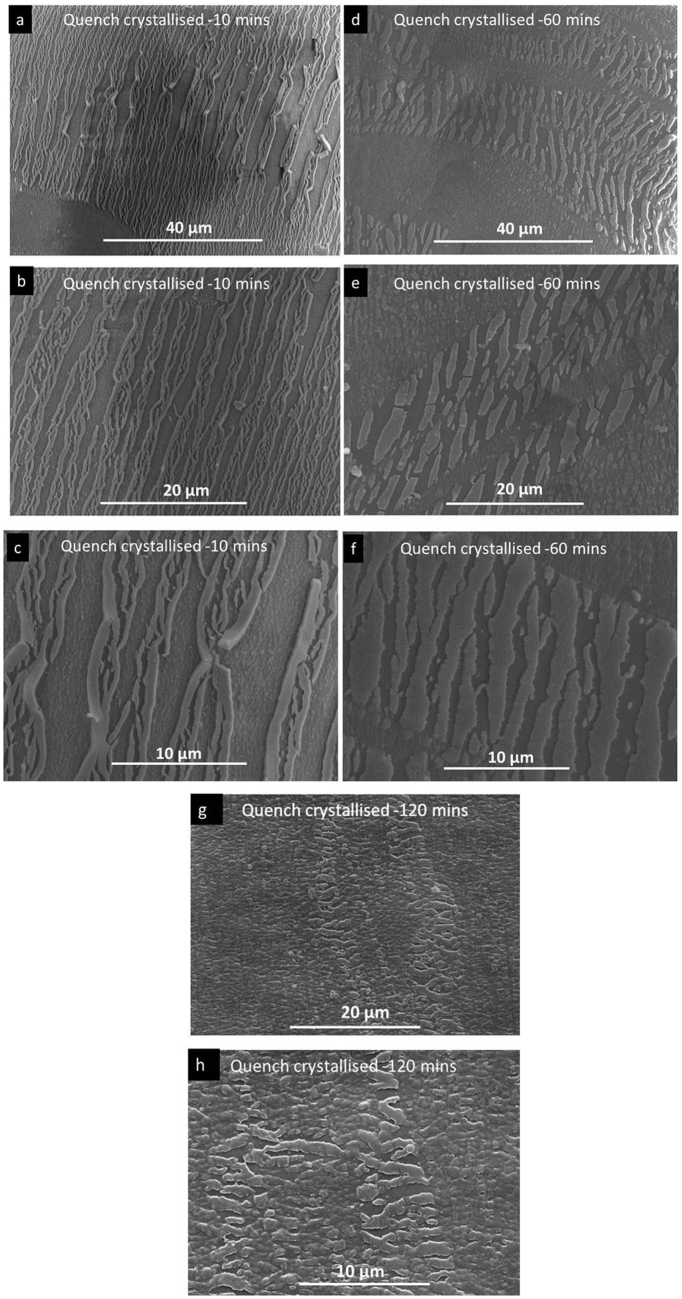
SEM image showing the fragmentation of the fibre-like crystal presented in quench crystallised PEEK film annealed for 10 mins (**a**–**c**), 60 mins (**d**–**f**) and 120 mins (**g**–**h**) at different magnifications, respectively.

Detailed observation was further carried out within the necking region of the quench crystallised PEEK films annealed at 10, 60 and 120 mins. Figure 12(a) to (h) shows the fragmentation of the fibre-like crystals at various magnifications. The annealing time has great effect on the pattern of fragmentation. The samples annealed for 10 mins have a unique pattern of cleavage, the crystal splits into thin fibrils of approximately 100–500 nm thickness along its length. As the annealing time increases to 60 and 120 mins, the fragmentation pattern changes, the cleaved fibrils become thicker crystal blocks of approximately 1–3 µm thickness. These results suggest that the fracture of the fibrils and crystal blocks distribute the tension energy and therefore enhanced the ductility of quenched crystallised PEEK film.

**Figure 13:**
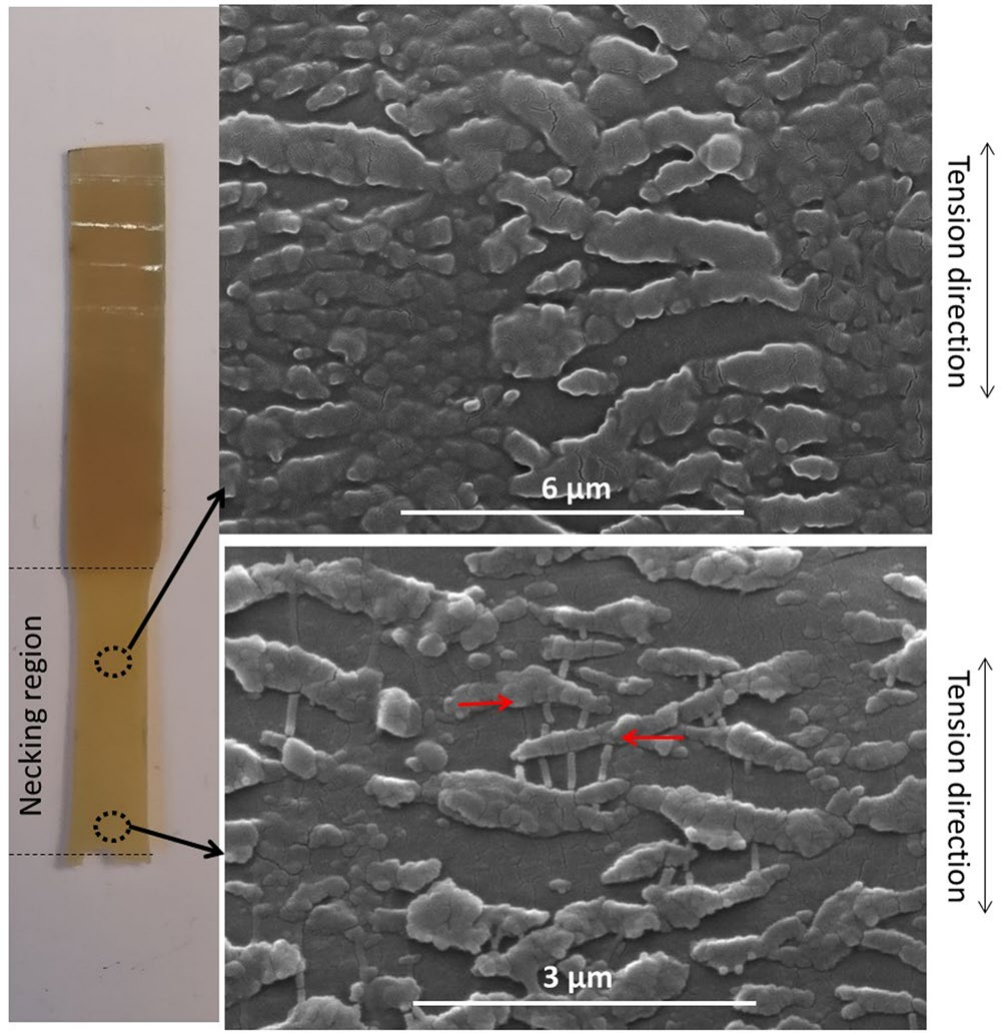
High magnification SEM images of fibre-like crystals annealed at 120 min presented at two different necking points.

The SEM images in Fig. 13, suggest that the fragmentation behaviour of fibre-like crystals in the necking region is different depending on the distance to the fracture point. The fibre-like crystals which are farther away from the fracture end are only split into large crystal blocks of approximately 500 nm. Closer to the break point, the crystal blocks seems to split further into thinner strips bridged together by thin fibrils aligned in the tension direction, almost perpendicular to the crystal strips. The connecting fibrils are marked with red arrows in Fig. 13.

**Figure 14:**
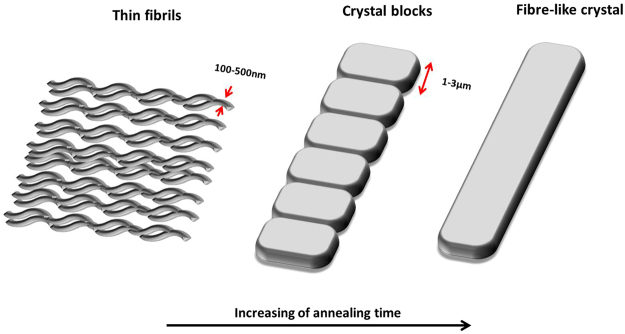
Schematic illustration of the fibre-like crystal evolution with annealing time.

Based on the SEM observations, a schematic illustration of the fibre-like crystal evolution with annealing time is proposed and shown in Fig. 14. The first stage involved in the development of fibre-like crystals is the formation of thin fibrils, the smallest structure observed in this study. However, the possibility of a submicron crystal structure inside these thin-fibrils can not be excluded. Our previous study has shown that the finest building unit of spherulitic PEEK is a primary granular crystal with the size approximately 20–30 nm. With increasing annealing time, these fibrils gradually merged along the fibre length to develop thicker compact crystal blocks. It is suspected that upon further increasing of annealing time, merging of blocks occurrs and one continuous piece of fibre-like crystal is formed. Based on this hypothesis, the fragmentation pattern of fibre-like crystals depends on the stage of crystallization.

## Conclusion

The PEEK thin films obtained through quenched crystallisation method revealed formation of a fibre-like crystals structure. XRD and TEM results indicate that these structures are a surface feature. The films presenting these fibre-like crystals on the surface exhibited an enhanced ductile behaviour reaching in some cases a maximum elongation at break of 70 to 90%, 3.5 to 4.5 times higher than that of the conventional spherulitic structure. DSC analysis suggests the crystallinity of PEEK film with fibre-like crystals is similar as the one with spherulitical crystals. This is also proven by the TEM cross-sectional images of the two types of films (melt crystallised and quenched crystalised) which show a similar spherulitic structure in the depth of the film. The DSC results imply that the enhanced ductility is originated from the differences in crystal morphology (surface and depth) of two types of films rather than the differences in level of crystallinity. The evolution process of fibre-like crystals has been proposed according to the microstructure characterization of the surface. Being able to control the formation and orientation of the fibre-like structure could lead to materials with better performance or unique properties for a wider range of applications.

## References

[1] Lovinger AJ, Davis DD (1986). Solution crystallization of poly(ether ether ketone). Macromolecules.

[2] Lovinger AJ, Davis DD (1985). Electron‐microscopic investigation of the morphology of a melt‐crystallized polyaryletherketone. J. Appl. Phys..

[3] Chung JS, Cebe P (1992). Morphology of poly(phenylene sulphide) single crystals grown by a two-stage self-seeding technique. Polymer.

[4] Vaughan AS, Bassett DC (1988). Early stages of spherulite growth in melt-crystallized polystyrene. Polymer.

[5] Uemura A (1986). Morphology of Solution-grown Crystals and Crystalline Thin FiIms of Poly (p-phenylene sulfide). Bull. Inst. Chem. Res..

[6] Uemura A, Tsuji M, Kawaguchi A, Katayama K (1988). High-resolution electron microscopy of solution-grown crystals of poly (p-phenylene sulphide). J. Mater. Sci..

[7] D’Ilario L, Piozzi A (1989). Poly(*p*-phenylene sulphide) single crystals. J. Mater. Sci. Lett..

[8] Medellin-Rodriguez FJ, Phillips PJ (1996). Bulk crystallization of poly(Aryl Ether Ether Ketone)(PEEK). Polym. Eng. Sci..

[9] MedellinRodriguez FJ, Phillips PJ (1990). Crystallization and Structure-Mechanical Property Relations in PEEK. Polym. Eng. Sci..

[10] Waddon AJ, Hill MJ, Keller A, Blundell DJ (1987). On the crystal texture of linear polyaryls (PEEK, PEK and PPS). J. Mater. Sci..

[11] Wang Y, Chen B, Evans KE, Ghita O (2016). Novel Fibre-like Crystals in Thin Films of Poly Ether Ether Ketone (PEEK). Mater. Lett..

[12] Wang Y, Beard JD, Evans KE, Ghita O (2016). Unusual crystalline morphology of Poly Aryl Ether Ketones (PAEKs). RSC Adv..

[13] Chivers RA, Moore DR (1994). The effect of molecular weight and crystallinity on the mechanical properties of injection moulded poly(aryl-ether-ether-ketone) resin. Polymer.

[14] Lee YC, Porter RS (1986). Crystallization of poly(etheretherketone) (PEEK) in carbon fiber composites. Polym. Eng. Sci..

[15] Gplastics., High Density Polyethylene, https://www.gplastics.com/pdf/hdpe.pdf (2017).

[16] Materials Spotlight., The Properties of Nylon 12, https://www.cableorganizer.com/articles/materials-nylon12.html (2017)

[17] AMILAN® Nylon Resin, General properties of heat-resistant nylon resins CM1026 and CM3006, https://www.toray.jp/plastics/en/amilan/technical/tec_021.html (2017)

[18] Victrex, PEEK 150PF Datasheet. https://www.victrex.com/~/media/datasheets/victrex_tds_150pf.pdf (2017)

[19] Bassett DC, Olley RH (1988). and Al Raheil. On crystallization phenomena in PEEK. Polymer.

